# Features specific to retinal pigment epithelium cells derived from three-dimensional human embryonic stem cell cultures — a new donor for cell therapy

**DOI:** 10.18632/oncotarget.8185

**Published:** 2016-03-18

**Authors:** Wei Wu, Yuxiao Zeng, Zhengya Li, Qiyou Li, Haiwei Xu, Zheng Qin Yin

**Affiliations:** ^1^ Southwest Hospital/Southwest Eye Hospital, Third Military Medical University, Chongqing 400038, China; ^2^ Key Laboratory of Visual Damage and Regeneration and Restoration of Chongqing, Chongqing 400038, China

**Keywords:** retinal pigment epithelium cells, human embryonic stem cells, three-dimensional cultures, retinal degenerative diseases, cell therapy

## Abstract

Retinal pigment epithelium (RPE) transplantation is a particularly promising treatment of retinal degenerative diseases affecting RPE-photoreceptor complex. Embryonic stem cells (ESCs) provide an abundant donor source for RPE transplantation. Herein, we studied the time-course characteristics of RPE cells derived from three-dimensional human ESCs cultures (3D-RPE). We showed that 3D-RPE cells possessed morphology, ultrastructure, gene expression profile, and functions of authentic RPE. As differentiation proceeded, 3D-RPE cells could mature gradually with decreasing proliferation but increasing functions. Besides, 3D-RPE cells could form polarized monolayer with functional tight junction and gap junction. When grafted into the subretinal space of Royal College of Surgeons rats, 3D-RPE cells were safe and efficient to rescue retinal degeneration. This study showed that 3D-RPE cells were a new donor for cell therapy of retinal degenerative diseases.

## INTRODUCTION

Retinal pigment epithelium (RPE) is a monolayer of pigmented cells between the neural retina and the choriocapillaris. RPE can secrete nutrition factors, phagocytose photoreceptor outer segment (POS) and is involved in the formation of the blood-retinal barrier; the pigment granules in RPE cells have light-absorbing and anti-oxidative stress functions [1]. Dysfunction of RPE usually results in secondary degeneration of photoreceptor cells, thus causing sight-threaten diseases, including age-related macular degeneration (AMD) and Stargardt's macular dystrophy [2]. Unfortunately, no effective treatment is found for curing these retinal degeneration diseases currently. RPE cell transplantation is a promising approach and the clinical trials are underway [3–7].

Currently, in addition to direct isolation from allogeneic RPE, RPE donor cells can be obtained from embryonic stem cells (ESCs), induced pluripotent stem cells (iPS), and non-pluripotent stem cells [8]. Although the risk of immune rejection is inevitable, ESCs are indefinite self-renewing, better characterized and more economical requiring less genetic manipulation [9]. Therefore, human ESCs (hESCs) are considered as a potential donor source of RPE cells in clinical application. More recently, the hESC-derived RPE cells induced by spontaneous differentiation method (SD-RPE) have been used in patients with AMD and Stargardt's macular dystrophy [4, 5, 7], and the safety and efficiency have been preliminarily testified. However, to advance clinic translation, it's crucial to control the *in vitro* differentiation stage of RPE cells derived from hESCs to maximize vitality and functionality during and after transplantation [4, 5]. What's more, many concerns have yet to be determined, including whether spontaneous differentiation method is the most ideal approach to obtain donor RPE cells. In addition to the spontaneous differentiation method which allows the overgrowth or embryoid bodies of ESCs to achieve spontaneous differentiation of RPE cells [4, 10–13], there are two other approaches to propagate RPE cells derived from ESCs, either directed induction method or three-dimensional (3D) hESC cultures [3, 14, 15]. The former method uses defined factors to target the induction of ESCs into RPE cells [16–18]. The directed differentiation procedures, however, required very long culture times and, so far, did not show enough advancement compared to the spontaneous method to justify their application for therapeutic purposes [2, 14]. The recently reported 3D ESC cultures method, based on 3D embryoid body differentiation protocol, generates self-organizing optic cups mimicking normal development of embryonic retinal tissue [15, 19]. Subsequently, photoreceptors and retinal ganglion cells derived from 3D ESC or iPS cultures are comprehensively studied, which show a high similarity with *in-vivo* counterpart and many advantages over two-dimensional induction derivatives [20–23]. Thus 3D ESC cultures are expected to yield RPE cells that are equivalent to native RPE cells. Moreover, 3D hESCs cultures have the exclusive potential to simultaneously provide not only RPE cells but also other retinal cells, such as photoreceptors, for clinical application of stem cell based cell therapy. However, it has yet to determine the characteristics of RPE cells derived from 3D hESC cultures (3D-RPE).

Here, we optimized the generation of 3D-RPE cells and studied the time-course characteristics of 3D-RPE cells from the perspectives of cell morphology, pigment, ultrastructure, growth features, gene expression profiles, and cell functions.

## RESULTS

### The differentiation of hESCs toward RPE cells

**Figure 1:**
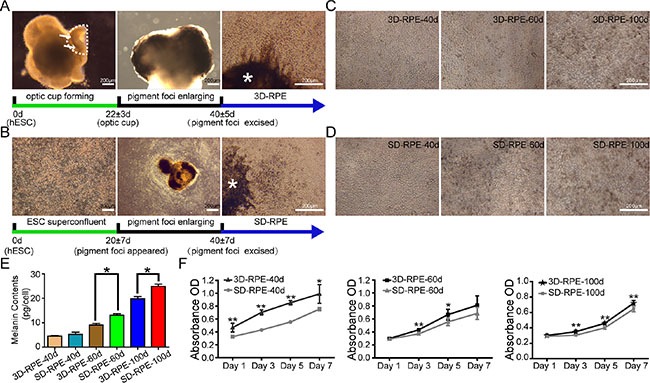
3D-RPE cells had lighter pigment but better proliferation than SD-RPE cells (**A**) Schematic of 3D-RPE cells differentiation protocol. By three-dimensional cultures, hESCs differentiated into 3D-RPE cells through optic cup self-organization, pigment foci enlarging and excised. Dotted line indicated the outline of optic cup like structure. Arrows indicated pigment. Asterisk indicated pigment foci. The cells spreading outwards from excised pigment foci were 3D-RPE cells. (**B**) Schematic of SD-RPE cells differentiation protocol. By two-dimensional cultures, hESCs differentiated into SD-RPE cells through super-confluence, pigment foci enlarging and excised. Asterisk indicated pigment foci. The cells spreading outwards from excised pigment foci were SD-RPE cells. (**C** and **D**) The phase contrast images of 3D-RPE and SD-RPE cells at day 40, day 60 and day 100 after differentiation. (**E**) Quantitative pigment of 3D-RPE and SD-RPE cells at day 40, day 60 and day 100 (bars: mean + s.d.; *n* = 3). Asterisk (*) indicated *p* value < 0.05. (**F**) The growth curve of 3D-RPE and SD-RPE cells at day 40, day 60 and day 100 by WST-8 assay (bars: mean ± s.d.; *n* = 6). Asterisk (*) indicated *p* value < 0.05. Double asterisk (**) indicated *p* value < 0.01.

After 3D culture for 19–25 days, hESC cells formed two-walled optic cup like structures, which were hemispherical in shape, with monolayer sheet of pigment on the outside (Figure 1A). Immunohistochemistry analysis confirmed that the inner layer of the optic cup tissues expressed the neural retina marker PAX6, and the outer pigment layer expressed the RPE cell-specific marker CRALBP (Supplementary Figure S1). Similar to the Ali's protocol [20], we kept the whole embryoid bodies for the entire period of 3D culture to produce abundant 3D-RPE cells. After optic cups were continuously cultured for three weeks, pigments gradually enlarged and formed pigment foci. At 35–45 days, these pigment foci were excised and were placed onto 6-well plates to allow 3D-RPE cells expanding. To study the time-course characteristics, the day when 3D-RPE cells spread outwards from pigment foci was designated as 1 day post-differentiation (Figure 1A). We used SD-RPE cells as control. By spontaneous differentiation, hESC colonies become super-confluent after two-dimensional differentiation for 6–8 days, and formed pigment foci about 10 days later. At 33–47 days, when pigment foci were large enough, they were excised and placed onto 6-well plates to allow SD-RPE cells expanding. Similar to above, this day was designated as 1 day of SD-RPE cells differentiation (Figure 1B).

Firstly, immunostaining of MITF and PAX6 was done to assess the purity of the RPE cultures at 10 days post-differentiation. It showed that near 100% of 3D-RPE and SD-RPE cells were both MITF and PAX6 positive (Supplementary Figure S2). Meanwhile absence of staining in OCT4 and Nanog indicated the 3D-RPE and SD-RPE cells did not mixed with pluripotent cells (data not shown). As early as 20 days of differentiation, 3D-RPE and SD-RPE cells became hexagonal. After 40 days of differentiation, pigment was not present in 3D-RPE and SD-RPE cells (Figure 1C and 1D). After 60 days of differentiation, 3D-RPE cells still did not have pigment, while SD-RPE cells already had marked pigment (Figure 1C and 1D). After 100 days of differentiation, both 3D-RPE and SD-RPE cells were pigmented (Figure 1C and 1D). Consistently, quantitative analysis showed that the pigment concentration in the 3D-RPE and SD-RPE cells increased gradually (Figure 1E). There was no significant difference between the pigment concentration of 3D-RPE-40d and SD-RPE-40d, whereas 3D-RPE cells had lighter pigment at 60 days and 100 days of differentiation compared with the time-matched SD-RPE cells respectively.

### Attachment and proliferation of 3D-RPE cells

To characterize the attachment and proliferation of 3D-RPE, we trypsinized confluent RPE cells and reseeded in 96-well plate. The growth of 3D-RPE and SD-RPE cells at 1, 3, 5, and 7 days after inoculation were detected using the WST-8 assay. The absorbance values after 1 day of inoculation were used to define attachment capacities and the absorbance values at other time points were used to evaluate the proliferation capacities.

After 1 day of inoculation, it showed that 3D-RPE-40d possessing an optimal attachment capacity whereas the absorbance values of the other cell types were not significantly different (Figure 1F). As for the cell proliferation, it showed that at all differentiation time points, the proliferation capacities of 3D-RPE cells were all significantly higher than that of time-matched SD-RPE cells; as differentiation advanced, the proliferation capacities of these two cell types both gradually decreased.

### Ultrastructure of 3D-RPE cells

**Figure 2:**
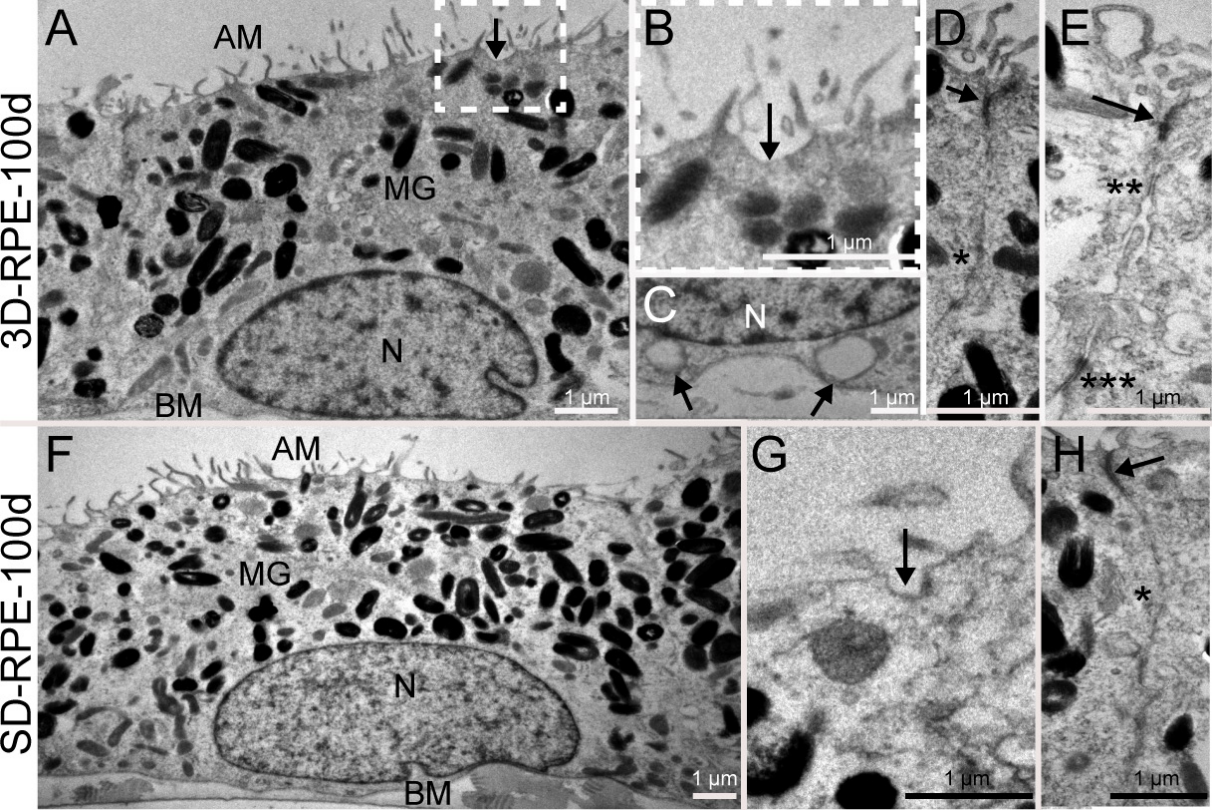
The ultrastructure of 3D-RPE and SD-RPE cells (**A**) 3D-RPE-100d cells had abundant apical microvilli (AM), melanin granules (MG) in the apical regions and the nucleus (N) in the basolateral regions. A basement membrane (BM) was visible. (**B**) A higher magnifications of the areas framed in Figure 2A showed caveolae (arrow) was distributed on the apical plasmalemma. (**C**) Caveolaes (arrow) were also distributed on the basolateral plasmalemma. (**D**) Tight junction (arrow) and gap junction (single asterisk) could be found. (**E**) Adherences junction (double asterisk) and desmosome (triple asterisk) could be found. (**F**) SD-RPE-100d cells had also abundant AM, MG in the apical regions and the nucleus (N) in the basolateral regions. A BM was visible. (**G**) Caveolae (arrow) was distributed on the apical plasmalemma in SD-RPE-100d cells. (**H**) Tight junction (arrow) and gap junction (single asterisk) could be found.

The ultrastructure of 3D-RPE was detected using transmission electron microscopy (TEM). After 100 days of differentiation, microvilli were apparent at the apices of 3D-RPE, pigment granules were accumulated in the apical cytoplasm, and nuclei were located at the basal part (Figure 2A). We also found that in 3D-RPE-100d cells, caveolaes occurred at both the apex and the base of the cell membrane, which contributed to the metabolism of the visual cycle and the compartmentalization of cell signaling [24] (Figure 2B and 2C). Besides, all of the intercellular connections specific to RPE cells existed including tight junction, gap junction, adherences junction and desmosomes (Figure 2D and 2E). As a control, SD-RPE-100d cells also showed abundant microvilli, apical pigment granules and basal nuclei (Figure 2F). In line with our previous pigment quantitation results, there were markedly more pigment granules in SD-RPE-100d cells. However, in SD-RPE-100d cells, caveolae only occurred at the cell membrane apices (Figure 2G) and only tight junction and gap junction between adjacent cells were detected (Figure 2H).

### Transcriptome analyses of 3D-RPE cells

**Figure 3:**
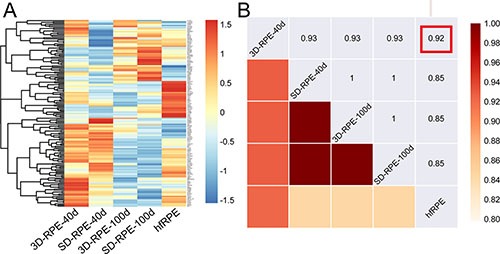
The RPE signature genes expression profile of 3D-RPE and SD-RPE cells (**A**) Heat map of 154 genes selected as RPE signature genes in 3D-RPE-40d, SD-RPE-40d, 3D-RPE-100d, SD-RPE-100d and native hfRPE. Normalized expression levels were shown in a blue-white-red gradient. (**B**) Spearman correlation matrix between the five groups, measured by gene expression levels of RPE signature genes. Red frame indicated 3D-RPE-40d had the highest correlation with hfRPE compared with other RPE cells.

To comprehensively study 3D-RPE cells, we examined expression profile of the 154 RPE signature genes in 3D-RPE-40d and 3D-RPE-100d [25]. SD-RPE-40d, SD-RPE-100d and native human fetal RPE (hfRPE) served as control since hfRPE was a well-regarded differentiation model [26, 27]. Figure 3A showed a heatmap of normalized expression levels of each group. Combing with Spearman correlation analysis, we found that 3D-RPE-40d had the highest correlation with native hfRPE, and that 3D-RPE-100d, SD-RPE-40d and SD-RPE-100d shared similar expression levels of 154 RPE signature genes (Figure 3B).

### The differentiation and maturation of 3D-RPE cells

**Figure 4:**
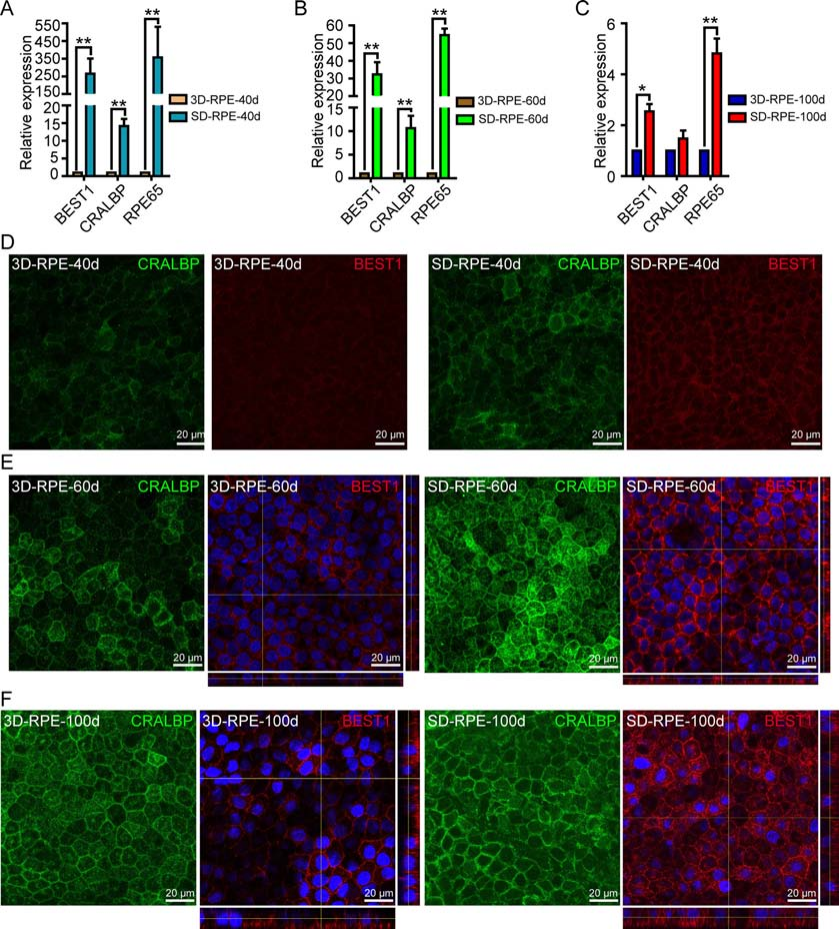
The differentiation of 3D-RPE cells (**A–C**) RT-qPCR analysis of expression of CRALBP, BEST1 and RPE65 in 3D-RPE cells versus time-matched SD-RPE cells at day 40, day 60 and day 100 after differentiation respectively (bars: mean + s.d.; *n* = 3). All samples were normalized to glyceraldehyde 3-phosphate dehydrogenase (GAPDH). Single asterisk (*) indicated *p* value < 0.05 and double asterisk (**) indicated *p* value < 0.01. (**D–F**) Immunostaining of CRALBP and BEST1 of 3D-RPE and SD-RPE cells at day 40, day 60 and day 100 after differentiation respectively. Z-stack fluorescent images with cross-section side views were displayed to show BEST1 location.

To determine the time-course status of differentiation and maturation in 3D-RPE, we detected the expression of maturation markers, including BEST1, CRALBP, and RPE65. Using RT-qPCR experiments, we found that the expression levels of BEST1, CRALBP, and RPE65 in 3D-RPE cells increased gradually as differentiation proceeded. However, the genes expression levels were significantly lower in 3D-RPE cells than those in time-matched SD-RPE cells and the gap of the genes expression levels between these two kinds of cells narrowed gradually (Figure 4A–4C). As expected, immunohistochemistry confirmed the genes expression pattern. After 40 days of differentiation, 3D-RPE-40d cells expressed barely detectable levels of CRALBP and BEST1 (Figure 4D). Subsequently, 3D-RPE-60d and 3D-RPE-100d cells showed stronger expression of CRALBP and BEST1. What's more, using Z-stack fluorescent images with cross-section side views, polarized expression of BEST1 were observed in 3D-RPE-100d cells (Figure 4E–4F). As a control, SD-RPE cells showed stronger expression of CRALBP and BEST1 than time-matched 3D-RPE cells, and SD-RPE-60d cells begun to show polarized expression of BEST1 (Figure 4D–4F). In addition, western blot and immunohistochemistry assays proved similar expression pattern of RPE65 in 3D-RPE and SD-RPE cells (Supplementary Figure S3).

In contrast, PAX6, as a RPE precursor marker gene, showed opposite tendency during the differentiation and maturation of 3D-RPE. By immunohistochemistry assay, it showed that the level of PAX6 in 3D-RPE cells decreased gradually as differentiation proceeded. However, the expression of PAX6 in 3D-RPE cells was significantly higher than that in time-matched SD-RPE cells (Supplementary Figure S4). Taken together, it suggested that 3D-RPE cells would mature progressively as differentiation proceeded and 3D-RPE cells were less mature than time-matched SD-RPE cells.

### Phagocytic ability of 3D-RPE cells

**Figure 5:**
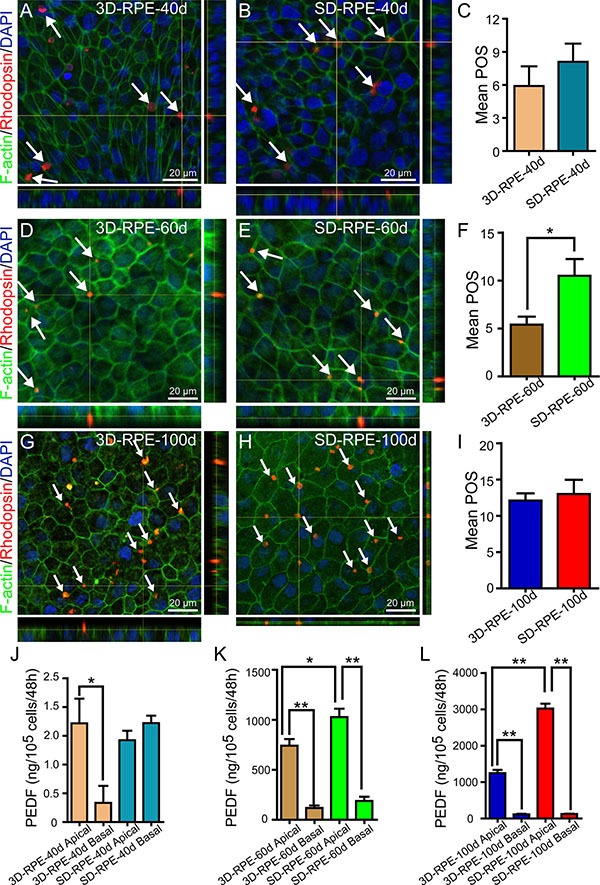
The functions of POS phagocytosis and PEDF secretion in 3D-RPE cells (**A–B**, **D–E**, **G–H**) Z-stack fluorescent images with cross-section side views showed the location of internalized POS in 3D-RPE and SD-RPE cells at day 40, day 60 and day 100. POS was immunostained by Rhodopsin (red). F-actin was stained using phalloidin (green) to show the cell morphology. Nuclei were stained with DAPI (blue). Arrows indicated internalized POS. (**C, F, I**) The number of internalized POS by 3D-RPE and SD-RPE cells at day 40, day 60 and day 100 per field view (135 μm × 180 μm; bars: mean + s.d.; *n* = 3). Single asterisk (*) indicated *p* value < 0.05. (**J–K, L**) ELISA analysis of PEDF secretion by 3D-RPE and SD-RPE cells at day 40, 60 and 100 (bars: mean + s.d.; *n* = 3). Single asterisk (*) indicated *p* value < 0.05. Double asterisk (**) indicated *p* value < 0.01.

Neural retinas of rats were peeled and co-cultured with RPE cells for 48 h to detect the capacity to phagocytize POS. To verify the POS was internalized in cytoplasm, orthogonal views of stacking images were used with immunostaining of Rhodopsin and F-actin labeling POS and cytoskeleton respectively. As expected, 3D-RPE cells could phagocytize POS on days 40, 60, and 100 (Figure 5A, 5D, 5G). Quantitative analysis showed that phagocytic ability of 3D-RPE cells enhanced gradually (Figure 5C, 5F, 5I). By comparison, 3D-RPE cells phagocytized as many POS as SD-RPE cells did on days 40 and 100 (Figure 5C and 5I). However, SD-RPE-60d phagocytized significantly more POS than 3D-RPE-60d did (Figure 5F).

### Pigment epithelium–derived factor (PEDF) secretion of 3D-RPE cells

PEDF, a nutrition factor with multiple retina protection functions, is firstly identified in the conditioned media of human fetal and adult RPE cell cultures [28]. To detect the PEDF secretion capacities of 3D-RPE cells, the PEDF gene expression level was measured with RT-qPCR assay. It confirmed that PEDF upregulated gradually in 3D-RPE cells as differentiation proceeded but was lower than that in time-matched SD-RPE cells (Supplementary Figure S5). Consistently, ELISA verified the similar PEDF expression pattern from protein level (Figure 5J–5L). In addition, we found polarized PEDF secretion in 3D-RPE cells as early as 40 days after differentiation whereas SD-RPE cells secreted polarized PEDF since 60 days after differentiation.

### Tight junction of 3D-RPE cells

**Figure 6:**
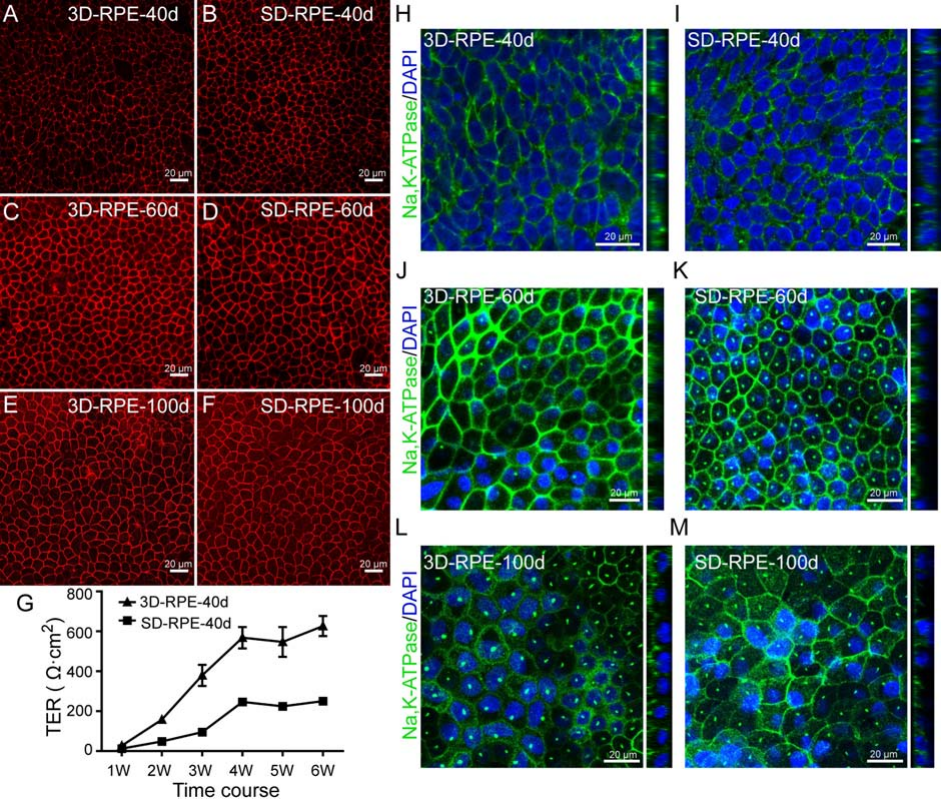
The tight junction and polarity in 3D-RPE cells (**A–F**) ZO-1 immunofluorescence staining of 3D-RPE and SD-RPE cells at day 40, day 60 and day 100 after differentiation. (**G**) TER measurements of 3D-RPE and SD-RPE cells (bars: mean + s.d.; *n* = 4). (**H–M**) The location of Na, K-ATPase in 3D-RPE and SD-RPE cells at day 40, day 60 and day 100 after differentiation.

To characterize the tight junction of 3D-RPE cells, we performed qualitative and quantitative assays. Through a robust ZO-1 immunostaining, we verified that 3D-RPE cells formed tight junction on day 40, 60 and 100 (Figure 6A, 6C, 6E). Furthermore, functional transepithelial electrical resistance (TER) measurements showed a time-dependent increase in 3D-RPE cells (Figure 6G). After being cultured for 6 weeks on Transwell membranes, the TER of 3D-RPE cells reached 627 ± 50 (Ω cm^2^). Although SD-RPE cells also showed a robust ZO-1 immunostaining on day 40, 60 and 100 (Figure 6B, 6D, 6F), the TER of 3D-RPE cells was significantly higher than that of time-matched SD-RPE cells (Figure 6G), indicative of a better epithelial barrier function in 3D-RPE cells.

### The polarity of 3D-RPE cells

The RPE monolayer is naturally polarized *in vivo*, which is of great importance for normal function of RPE. In addition to polarized location of BEST1 and secretion of PEDF described above, we detected Na, K-ATPase immunostaining since Na, K-ATPase was localized apically *in situ* and was believed to be associated to the process of phototransduction [29]. On day 40, Na, K-ATPase was expressed at the apical membrane in 3D-RPE cells (Figure 6H). However, Na, K-ATPase became nonpolar in SD-RPE-40d (Figure 6I). On days 60 and 100, Na, K-ATPase was expressed at the cell apex in both cell types (Figure 6J–6M). Taken together, our results suggested 3D-RPE cells could form excellent polarized monolayer.

### The gap-junctional intercellular communication (GJIC) of 3D-RPE cells

**Figure 7:**
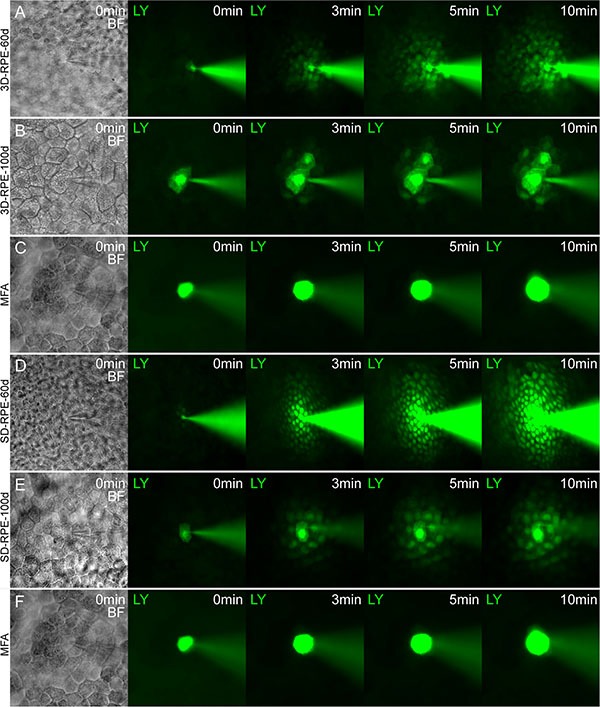
Gap junctional intercellular communication in 3D-RPE cells (**A–B, D–E**) Lucifer yellow (LY) transfer in adjacent 3D-RPE cells and SD-RPE cells at day 60 and day 100 after differentiation. (**C, F**) Specific gap junction blocker MFA inhibited LY transfer in adjacent 3D-RPE and SD-RPE cells.

Gap junction allows intercellular communication between adjacent RPE cells, which contributes to protect against oxidative stress [30]. In addition to the gap junction ultrastructure detected by TEM, we also investigated the GJIC in 3D-RPE cells by Lucifer Yellow (LY) transfer assay. After 40 days of differentiation, 3D-RPE cells could not transfer LY from one cell to another (data not shown). However, at day 60, 3D-RPE cells had a robust GJIC as shown in LY transfer assay (Figure 7A). At day 100, the LY transfer between adjacent 3D-RPE cells tended to lessen (Figure 7B). By comparison, LY transfer extent in 3D-RPE cells was smaller than that in time-matched SD-RPE cells (Figure 7D and 7E). A dramatic reduction in LY transfer by specific gap junction blocker MFA proved the material transport was mediated by gap junction (Figure 7C and 7F).

### The safety of 3D-RPE cells

To test whether 3D-RPE cells were safe enough for clinical application, we subcutaneously injected 3D-RPE-40d (*n* = 7), 3D-RPE-60d (*n* = 7) and 3D-RPE-100d cells (*n* = 6) into severe combined immune deficiency (SCID) mice when hESCs (*n* = 6) were used as a positive control. Importantly, we did not find any teratoma formed in any 3D-RPE cells transplanted mice in 4-month follow-up. By contrast, teratomas were observed in the hESCs group at 1 month post injection (data not shown).

### 3D-RPE cells could rescue retinal degeneration in Royal College of Surgeons (RCS) rats

**Figure 8:**
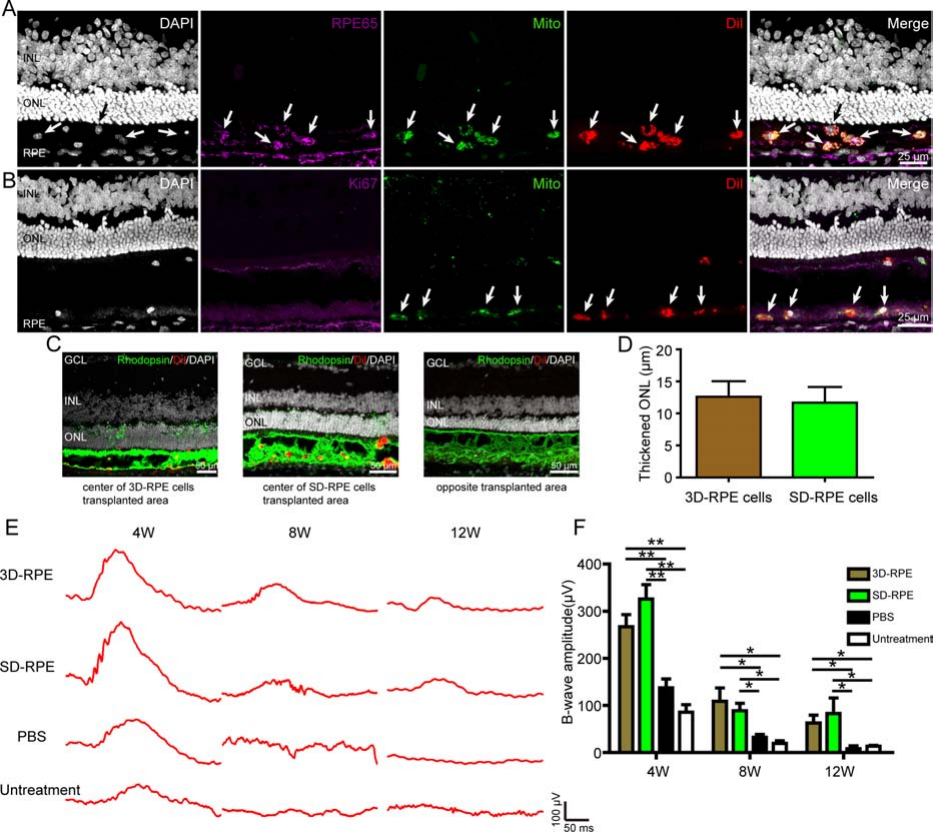
3D-RPE cells rescued retinal degeneration when grafted into the subretinal space of RCS rats (**A**) Immunofluorescence staining showed colocalisation of human mitochondria and RPE65 in Dil-labeled transplanted 3D-RPE cells. Arrows indicated viable transplanted 3D-RPE cells which were human mitochondria, RPE65 and Dil positive. (**B**) Immunofluorescence staining showed the absence of Ki67 in transplanted 3D-RPE cells. Arrows indicated viable transplanted 3D-RPE cells with human mitochondria and Dil positive. (**C**) Representative immunofluorescence images of Rhodopsin in central transplanted area of 3D-RPE cells and SD-RPE cells and opposite transplanted area. (**D**) Thickened ONL (ONL thickness of central transplanted area subtracted ONL thickness in opposite transplanted area) of 3D-RPE and SD-RPE cells (bars: mean + s.d.). (**E**) Representative ERG traces at 4, 8 and 12 weeks after transplantation. (**F**) The mean B-wave amplitudes at 0 db showed similar visual function rescue after 3D-RPE and SD-RPE cells transplantation (bars: mean + s.d.). Single asterisk (*) indicated *p* value < 0.05. Double asterisk (**) indicated *p* value < 0.01. Abbreviations: outer nuclear layer (ONL); inner nuclear layer (INL); ganglion cell layer (GCL).

To further validate if 3D-RPE cells can rescue retinal degeneration *in vivo*, we transplanted 3D-RPE-60d cells into the subretinal space of RCS rats at postnatal day 21 (P21), a well-acknowledged retinal degeneration model due to phagocytosis defect in the RPE [31, 32]. Meanwhile, SD-RPE-60d cells served as a control. In order to identify transplanted cells, we labeled 3D-RPE-60d and SD-RPE-60d cells with Dil before transplantation, and performed histological immunostaining of human specific marker (human mitochondria), RPE specific maker (RPE65) and Ki67 in frozen sections of enucleated eyes. It showed that 4 weeks after transplantation, grafted 3D-RPE-60d cells could form monolayer and could co-express RPE65 and human mitochondria (Figure 8A). Notably, 3D-RPE-60d cells were negative for RPE65 before transplantation. It suggested that 3D-RPE-60d cells could survive and mature gradually *in vivo*. However, some grafted cells clumped together in the subretinal space. The absence of Ki67 immunostaining suggested transplanted 3D-RPE-60d cells were quiescent 4 weeks post surgery (Figure 8B). Furthermore, we found the outer nuclear layer (ONL) thickness in regions with organized structure overlying transplanted 3D-RPE-60d cells was thicker than that in opposite regions without transplanted cells (Figure 8C). In order to compare the ONL protective effect of 3D-RPE-60d versus SD-RPE-60d cells, we calculated thickened ONL to minimize the individual differences. It showed that 4 weeks after transplantation, 3D-RPE-60d and SD-RPE-60d cells had similar protective effect on ONL thickness (Figure 8D). Finally, we used electroretinogram (ERG) assay to test the visual function at 4 weeks, 8 weeks and 12 weeks after transplantation. It showed that the amplitude of B wave in 3D-RPE-60d cells group was significantly higher than that in PBS and untreated group (Figure 8E and 8F). A). Over time, there was a reduction in acuity response for all the cell groups. Notably, the amplitude of B wave in 3D-RPE-60d and SD-RPE-60d cells were not significantly different.

## DISCUSSION

In this study, we got a new donor for cell therapy of retinal degenerative diseases, the 3D-RPE cells, and characterized 3D-RPE cells as Nazari's criteria [33].

Lanza et al. have reported that the pigmentation of RPE cells correlates with the differentiation status; higher pigmented RPE cells are of a more mature differentiation status with worse attachment and growth [4]. Consistently, we showed that 3D-RPE cells matured gradually with increasing pigment and decreasing proliferation as differentiation proceeded. Compared with SD-RPE cells, 3D-RPE cells were less pigmented and less mature with better proliferation and plasticity. Besides, we verified that 3D-RPE cells possessed specific RPE functions, such as POS phagocytosis, polarized PEDF secretion and tight junction, which would get better when they matured gradually. For reference, the TER of native hfRPE is reported to be 206 ± 151 (Ω cm^2^) [34]. As early as 3 weeks after seeding on Transwell filters, the TER of 3D-RPE cells was 379 ± 55 (Ω cm^2^) whereas the TER of SD-RPE cells reached 246 ± 5 (Ω cm^2^) after 4 weeks culture. It suggested that 3D-RPE cells formed native RPE-like confluent monolayer earlier. What's more, polarized Na, K-ATPase expression and PEDF secretion proved that 3D-RPE cells could form polarized monolayer earlier than SD-RPE cells.

By TEM observations, we found that 3D-RPE cells exhibited all of the characteristics of native RPE cells. The intercellular connections in 3D-RPE cells had important functions in the formation of the blood-retinal barrier, cell polarity, and intracellular communication [2]. Although SD-RPE cells also formed some characteristic ultrastructure of native RPE cells, we did not detect bipolar location of caveolaes and intercellular structures such as adherences junction and desmosomes.

Furthermore, 154 RPE signature genes expression profile verified that 3D-RPE-40d were the closest to native hfRPE. Due to the high correlation among 3D-RPE-100d, SD-RPE-40d and SD-RPE-100d cells, we inferred that 3D-RPE-100d was as mature as SD-RPE-40d. Giving the heterogeneity between each batch of differentiation, we performed 3 independent differentiation experiments to verify the differences between 3D-RPE and SD-RPE cells.

Since the maturation of 3D-RPE cells accompanied with decreasing proliferation and increasing functions, we suggested that medial stage of the 3D-RPE cells (3D-RPE-60d cells), which had moderate proliferation and robust functions of RPE cells, were ideal donor cells. As a result, we transplanted 3D-RPE-60d cells into the subretinal space of RCS rats. Importantly, 3D-RPE-60d cells did not show abnormal proliferation and could mature gradually *in vivo*. Moreover, 3D-RPE-60d cells transplantation caused better acuity response than PBS and untreatment group in ERG test. It suggested that 3D-RPE-60d cells delayed retinal degeneration for 12 weeks. However, we did not found 3D-RPE-60d cells had any superiority of rescuing retinal degeneration by comparing the ONL thickness and ERG assays with SD-RPE-60d cells. Consistently, Lanza et al. compared the effects of hESC-RPE cells with low, medium and high pigmentation when grafted into the subretinal space of RCS rats with a behavior test (spatial visual acuity), and no significant differences were observed [10]. Nevertheless, 3D ESC cultures hold the advantage of simultaneously yielding different donor cells for cell therapy and better proliferation of 3D-RPE cells means yielding more donor cells.

Taken together, we found that 3D-RPE cells were new donor for cell therapy, which resembled native hfRPE closely owing to 3D cultures mimicking normal development of embryonic retinal tissue. Compared with SD-RPE cells, 3D-RPE cells were less mature and had advantages in proliferation, ultrastructure, tight junction and polarity. However, whether 3D-RPE cells have differences with SD-RPE cells *in vivo* requires more studies.

## MATERIALS AND METHODS

### Ethics statement

All the animals used in this research were obtained from the Third Military Medical University and were maintained at pathogen-free conditions. All procedures were done according to protocols approved by the Institutional Review Board of the Southwest Hospital, Third Military Medical University and conformed to the NIH guidelines on the ethical use of animals. All experiments involving in human cells and tissues were carried out in accordance with the Tenets of the Declaration of Helsinki and were approved by the ethics committee of Southwest Hospital, Third Military Medical University.

### Differentiation of 3D-RPE and SD-RPE cells

In this study, we used hESC line H1, which was a gift from Shanghai Institute of Biochemistry and Cell Biology. The cell line had been tested and authenticated by immunostaining and teratoma formation assay in Shanghai Institute of Biochemistry and Cell Biology. One month later, we obtained the cell line and started this research. The hESC line H1 was maintained in commercially available mTESR^™^1 medium (05850, Stem Cell Technologies) without feeders.

For 3D-RPE differentiation, hESCs aggregates were cultured in serum-free and growth-factor-reduced medium (SFEBq) as Sasai's protocol with slight modifications [15]. In brief, 9 × 10^3^ dissociated hESCs were quickly reaggregated in per V-bottomed conical well (PrimeSurface 96V; Sumitomo Bakelite) with differentiation medium (G-MEM supplemented with 20% KSR, 0.1 mM nonessential amino acids, 1 mM pyruvate, 0.1 mM 2-mercaptoethanol) containing IWR1e (Merck) and Matrigel (growth-factor-reduced; BD Biosciences). On day 12, 96 aggregates were transferred to per 10 cm dish in differentiation medium supplemented with 10% FBS. From day 15 to day 18, 3 μM CHIR99021 (R & D) and 100 nM SAG (R & D) were added to differentiation medium. Since the day 18, the whole aggregates were cultured in DMEM/F12-Glutamax medium (GIBCO) containing the N2 supplement (Invitrogen), 10% FBS, 0.5 μM retinoic acid (Sigma-Aldrich) and 0.25 μg/mL Fungizone (GIBCO) without Activin. After longer culture of aggregates, massive pigment appeared gradually, and pigment foci were excised mechanically and were placed on 6-well plate coated with Matrigel (growth-factor-reduced; BD Biosciences).

The protocols for differentiation of SD-RPE were described previously [11]. Defining the day on which hESCs were cultured in basic hESC medium without bFGF as day 0, hESC colonies were allowed to become super confluent and formed pigment foci in 2∼3 weeks. When pigment foci had reached at least 1 mm in diameter, they were excised mechanically and were placed on 6-well plate coated with Matrigel (growth-factor-reduced; BD Biosciences).

When 3D-RPE and SD-RPE cells expanding from the pigment foci reached confluent in 6-well plate, they were passaged into a T25 flask for proliferation and purification. Then the RPE cells were cultured on Transwell filters (3470, Corning). The culture medium for RPE consisted of High glucose (4.8 g/L) Knockout Dulbecco's Modified Eagle's Medium (DMEM, Invitrogen), 20% Knockout serum replacement (KSR, Invitrogen), 1% non-essential amino acid solution, 1 mM L-Glutamine (Invitrogen) and 0.1 mM β-mercaptoethanol (Sigma).

### Measurement of melanin content

For measurement of melanin content, RPE cells pellets were counted by hemocytometer and then resuspended in 1N NaOH, denatured in 80°C for 10 minutes. A synthetic melanin (8631, Sigma) was serially diluted from 200 to 6.75 μg/mL as standards. Then the absorbance of samples and standards were measured at 475 nm by microplate reader (varioskan flash, Thermo) and the data was normalized to the total cell number extracted. Three repeats of each kind of cell were analyzed.

### WST-8 assay

For WST-8 assay, a Cell Counting kit-8 (CCK8, C0037, Beyotime) was used according to manufacturer's instructions. In brief, 100 μl of 3D-RPE or SD-RPE cells were seeded at 1 × 10^5^/mL in 96-wells plate. On the test day, the old medium was replaced with 100 μl of fresh cell medium and 10 μl CCK8 reagents. After incubated at 37°C, 5% CO_2_ for 1 h, the absorbance was measured at 450 nm by a microplate reader (varioskan flash, Thermo). Six repeats of each kind of cell were analyzed.

### Electron microscopy

3D-RPE-100d and SD-RPE-100d cells grown on Transwell filters were fixed in 2.5% glutaraldehyde in 0.1 M cacodylate buffer (pH 7.4) overnight. After washing with 0.1 M PBS twice, samples were post fixed with 1% osmium tetroxide for 2 h, washed with 0.1 M PBS, dehydrated with increasing concentrations of acetone, and embedded in epoxy resin 618. Ultrathin sections were cut using a diamond knife (Diatome, USA) and stained with 2% uranyl acetate and 2% lead citrate prior to examination in a JEM-1400 Plus (JEOL) electron microscope.

### Digital gene expression profile

Total RNA was extracted from 3D-RPE-40d, 3D-RPE-100d, SD-RPE-40d, SD-RPE-100d and native human fetal RPE (hfRPE) using Trizol (Invitrogen). The human fetal eyes (gestation, 13 weeks) were obtained from Southwest Hospital with ethics committee approval and donor informed consent. Then the native hfRPE was isolated as described previously [35]. Sequencing libraries were generated using NEBNext^®^ Ultra™ RNA Library Prep Kit for Illumina^®^ (NEB) following manufacturer's recommendations and library quality was assessed on the Agilent Bioanalyzer 2100 system. After cluster generation, the library preparations were sequenced on an Illumina Hiseq 2500 platform and 50 bp single-end reads were generated. For further details, see the SRA database (SRA: SRP061670). Reads Per Kilobase of exon model per Million mapped reads (RPKM) was used to estimate gene expression levels.

### Reverse transcription-quantitative polymerase chain reaction (RT-qPCR)

RT-qPCR experiments were performed as previously described [36]. In brief, total RNA was extracted using an RNAprep Pure Cell Kit (Sangon Biotech) according to the manufacturer's instructions. Total RNA (approximately 1–2 μg per 20 μl reaction) was reverse transcribed using a PrimeScript^®^ RT Reagent Kit (Takara). Quantitative PCR was performed by a CFX96 Real-Time PCR System (Bio-Rad) using a SYBR Green qPCR Mix (Dongsheng Biotech) according to the manufacturer's instructions. Relative expression levels were normalized to GAPDH and were calculated using the 2^−ΔΔC(t)^ method. All primers were listed in Supplementary Table S1.

### Phagocytosis of POS from rat retina

The phagocytosis assay was performed as previously described [37]. In brief, the retinas of non-dystrophic RCS rats at age 8 weeks were detached under dim red light. Photoreceptors of the isolated rat retinas were facing the RPE monolayers in Neurobasal A medium supplemented with 1% N2, 2% B27, 2 mM Glutamax (all from Gibco, Life Technologies), and 100 U/mL Penicillin/Streptomycin (Cambrex). After co-cultured with rat retinal explants for two days at 37°C, 5% CO_2_, the retinal explants were removed and 3D-RPE and SD-RPE cells were analyzed by immunostaining. The internalization of rat POS by RPE cells was immunostained using anti-Rhodopsin antibody (ab81702, 1:200, abcam), while filamentous actin was detected with FITC-conjugated phalloidin (P5282; Sigma-Aldrich) to demonstrate the cell morphology. In Z-axis scan model, we used Zen lite 2012 software and Zeiss LSM 700 confocal microscope to show the location of internalized POS. For quantitative calculation, 4 random fields of view (135 μm × 180 μm) were photographed per group and the number of internalized POS was counted. Three independent experiments were conducted.

### Enzyme linked immunosorbent assay (ELISA) for PEDF

The culture media in apical bath and basal bath from 3D-RPE and SD-RPE cells were collected 48 h after the previous medium was changed. Quantification of secretion levels was measured using PEDF Sandwich ELISA Kit (CYT420, Millipore) according to manufacturer's instructions. To assess the quantity of cells, samples were stained with DAPI and then photographed at 5 random locations (490 μm × 370 μm). Finally, the PEDF secretion levels were normalized to the quantity of 3D-RPE and SD-RPE cells. Three repeats of each kind of cell were analyzed.

### TER measurements

3D-RPE-40d and SD-RPE-40d cells were seeded at 1 × 10^5^ cells/cm^2^ on Transwell filters. After RPE cells formed hexagonal monolayers after 1 week, TER was measured using an EVOM epithelial voltohmmeter (World Precision Instruments, Hamden) according to manufacturer's instructions. The average of four Transwell filters with 3D-RPE or SD-RPE cells was used for further analysis. The TER of Empty Transwell filters only coated with Matrigel was measured as a control.

### Dye injection

Using the patch clamp technique, 1 μg/mL Lucifer Yellow (LY, L0259, Sigma) was injected into cytoplasm of single RPE cell. The intracellular perfusing solution contained 150 mM K-gluconate, 1 mM CaCl_2_, 4 mM MgCl_2_, 11 mM EGTA, 10 mM HEPES, pH 7.3 adjusted using KOH. The time-course lucifer yellow transfer was recorded by microscope. After three repeats per group, 30 μM meclofenamic acid (MFA, M4531-1G, Sigma) was incubated with cells for 10 min as a specific antagonist of gap junction. Then LY transfer between adjacent cells was analyzed by the same protocol.

### Teratoma formation

Teratoma formation protocol was according to our previous report [38]. Briefly, 100 μl of the cell suspension (1 × 10^6^ cells) was subcutaneously implanted into the inguina of SCID mice, regardless of sex. Twenty-six SCID mice were maintained in pathogen-free conditions at the animal facility of Third Military Medical University and received humane care according to the criteria outlined in the “Guide for the Care and Use of Laboratory Animals” prepared by the National Academy of Sciences. We monitored the tumor size and condition of the mice every week.

### Transplantation of RPE cells into RCS rats

Thirty-four RCS rats, P21, regardless of sex, were maintained in a 12 h light/dark cycle. 3D-RPE-60d and SD-RPE-60d cells were tested *in vivo*. After the animals were anesthetized using a mixture of 10 mg/kg ketamine (Sigma-Aldrich, St. Louis, MO, USA) and 1 mg/kg xylazine (Sigma-Aldrich), cell suspensions containing approximately 1 × 10^5^ cells in 1∼2 μl PBS were injected into the subretinal space through a small scleral incision with a fine glass pipette (Hamilton). The cornea was punctured to reduce intraocular pressure and to reduce the efflux of cells. A sham group was treated the same, except alone PBS was injected. The rats were immune-suppressed by addition of 210 mg/L cyclosporine A (Sandoz, Camberley) to the drinking water 24 h prior to the transplantation cells. The rats then remained on the same immunosuppressant throughout the experiment.

### Electroretinograms (ERG)

Full-field ERGs were recorded after overnight dark adaptation as our previous protocol [39]. In brief, rats were anesthetized and the pupils were dilated with Compound Tropicamide Eye Drops. Seventy eyeballs were tested, consisting of 3D-RPE cells group (*n* = 25), SD-RPE cells group (*n* = 23), PBS group (sham group, *n* = 10) and untreated group (*n* = 12). Then the rats were subjected to sequences of scotopic flashes of −20 and 0 db, with measurements averaged over five flashes. The resultant means of the maxima for A-wave and B-wave responses for the two sequences were analyzed.

### Immunostaining

Cells on Transwell filters or frozen sections were fixed with 4% paraformaldehyde for 15 min, permeabilized using 0.1% Triton X-100 in PBS for 15 min and blocked for 60 min in 5% goat serum. Primary antibodies against PAX6 (ab5790, 1:200, Abcam), BEST1 (ab14929, 1:400, Abcam), CRALBP (MA1813, 1:400, Thermo), RPE65 (ab13826, 1:100, Abcam), ZO-1 (40-2200, 1:100, Invitrogen), Na, K-ATPase (ab7671, 1:100, Abcam), Connexin43 (MAB3068, 1:200, Millipore), Rhodopsin (ab81702, 1:200, Abcam) and human mitochondria (ab3298, 1:500, Abcam: ab3598, 1:200, Millipore) were diluted in the same blocking buffer and incubated with the samples overnight at 4°C, then incubated with a fluorescently coupled secondary antibody 1 h at RT. The nuclei were stained with 4,6-diamidino-2-phenylindole (DAPI; Invitrogen). Fluorescence images were acquired with a confocal microscopy (Zeiss LSM 700, Carl Zeiss; software: Zen lite 2012). For quantification of PAX6 immunostaining, pictures were taken at 3 random locations and the percentage of positive stained cells was normalized to the number of DAPI stained nuclei inspected. For the ONL thickness analyses, random 3 RCS rats transplanted with 3D-RPE cells, SD-RPE cells and PBS respectively were quantified in central and opposite transplanted area using three serial sections at 100 μm intervals. To minimize the individual differences, normalized ONL protected effect (thickened ONL) was calculated by the ONL thickness in central transplanted area subtract the counterpart in opposite transplanted area.

### Statistical analysis

To compare two groups, data were analyzed by independent-samples *t* test. And multi-comparisons were done using One-Way ANOVA. We used SPSS software 17.0 and *P* < 0.05 was considered statistical significant.

## SUPPLEMENTARY MATERIALS FIGURES AND TABLE


