# Supplementary material for: Features specific to retinal pigment epithelium cells derived from three-dimensional human embryonic stem cell cultures — a new donor for cell therapy
Source: Oncotarget. 2016 Mar 18;7(16):22819–33. doi: 10.18632/oncotarget.8185 (PMC5008403; doi:10.18632/oncotarget.8185)
Supplement: Supplementary file 1 [file oncotarget-07-22819-s001.pdf]

Features specific to retinal pigment epithelium cells derived from three-dimensional human embryonic stem cell cultures — a new donor for cell therapy

Supplementary Materials

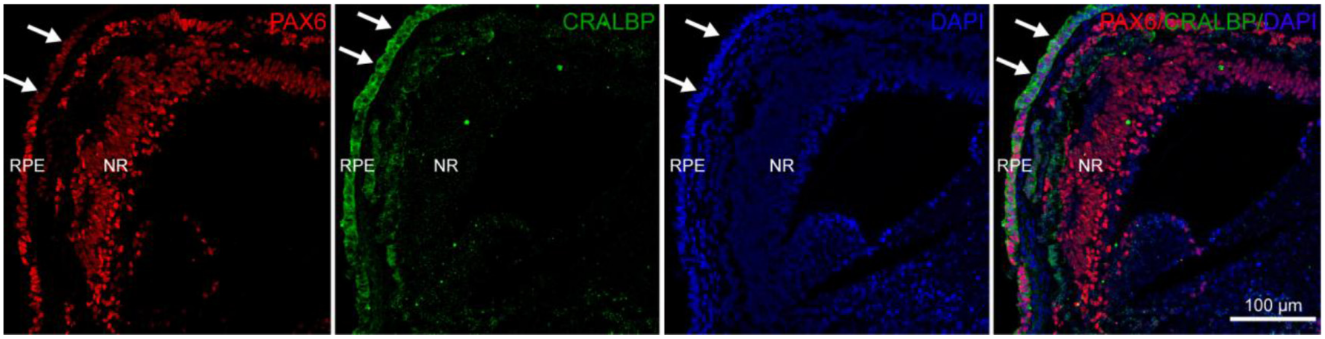

Supplementary Figure S1: The Immunostaining of optic cup. Abbreviations: neural retina (NR).

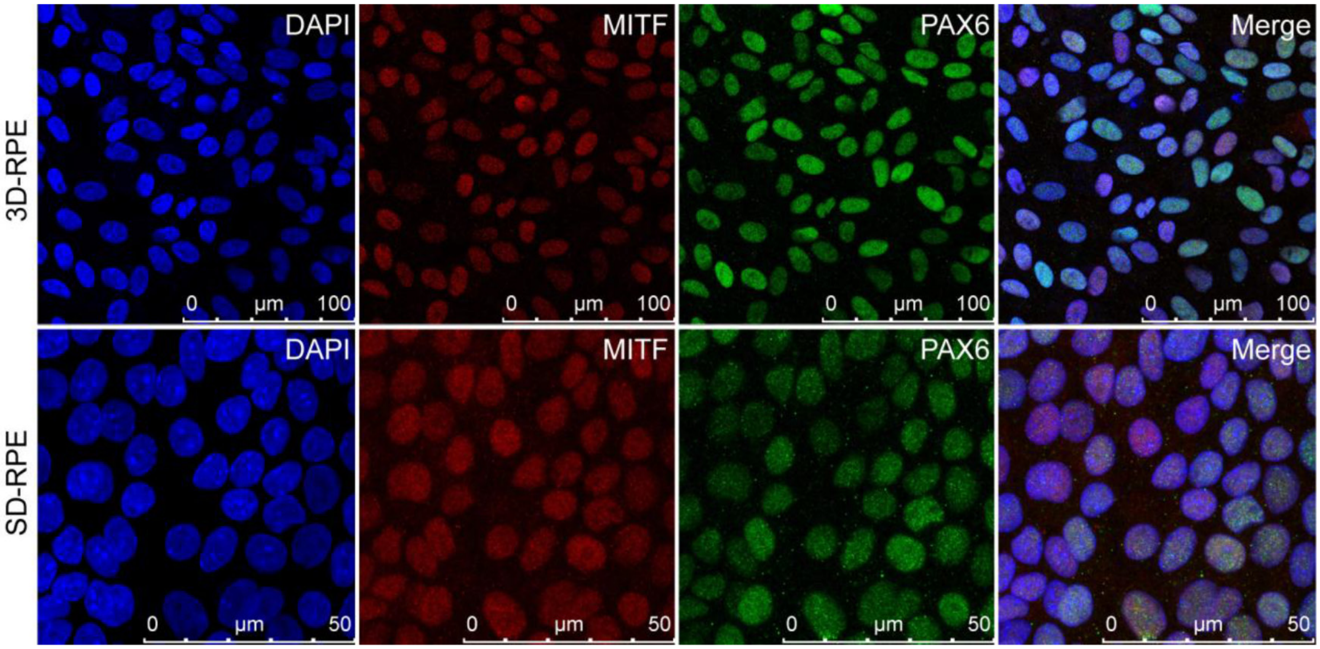

Supplementary Figure S2: The Expression of MITF and PAX6 in 3D-RPE and SD-RPE Cells.

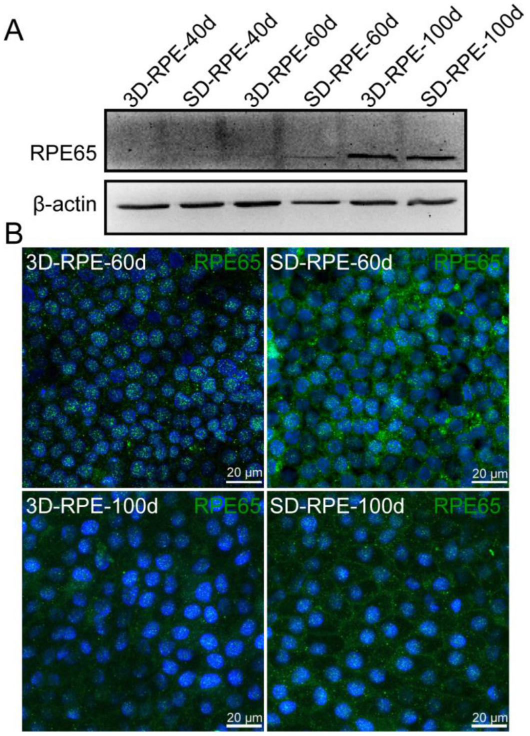

Supplementary Figure S3: The Expression of RPE65 in 3D-RPE and SD-RPE Cells. (**A**) Immunoblotting of RPE65 of 3D-RPE and SD-RPE at day 40, day 60 and day 100. (**B**) Immunofluorescence of RPE65 of 3D-RPE and SD-RPE at day 60 and 100 with nuclear staining with DAPI (blue).

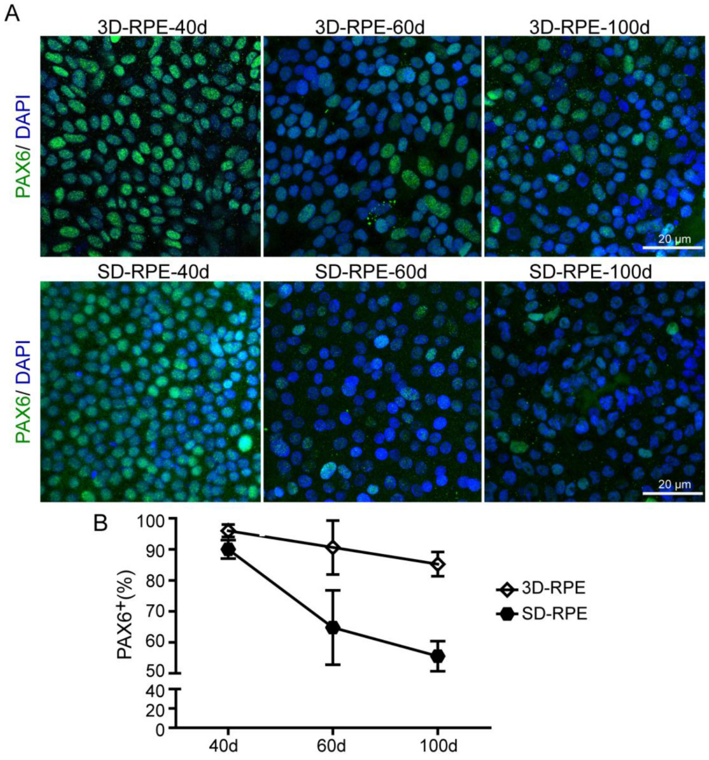

Supplementary Figure S4: The Expression of PAX6 in 3D-RPE and SD-RPE Cells. (**A**) Immunostaining of PAX6 (green) of 3D-RPE and SD-RPE at day 40, day 60 and day 100 with nuclear staining with DAPI (blue). (**B**) Percentage of positive PAX6 stained cells normalized to the number of DAPI stained nuclei (bars: mean ± s.d.).

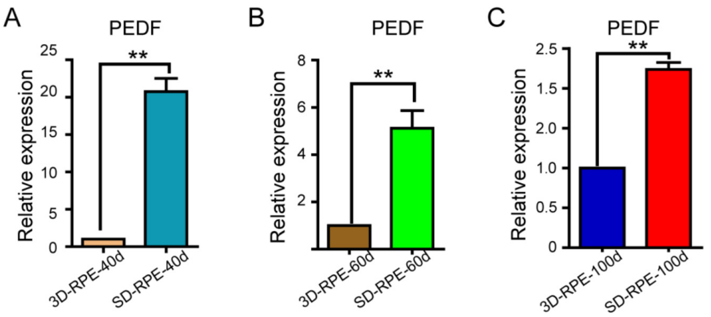

Supplementary Figure S5: The Expression of PEDF in 3D-RPE and SD-RPE Cells. (**A–C**) RT-qPCR analysis of PEDF expression in 3D-RPE and SD-RPE cells at day 40, 60 and 100 (bars: mean ± s.d.). GAPDH was used as an internal control. Double asterisk (**) indicated p value < 0.01.

Supplementary Table S1: Primers for RT-qPCR

Name	Sequence
BEST1-F	TCCCACCTGCCTAGTCGCCA
BEST1-R	TTCTAGATGCTGCCCCGCCA
CRALBP-F	CCCCGCCACACCTTGCAGAA
CRALBP-R	TTCCGTGCGCGGATGAAGCG
RPE65-F	CAGATGCCTTGGAAGAAGATG
RPE65-R	GGCATTCAGAATCAGGAGATAAG
PEDF-F	GGGAGCGGAGCAGCGAACAG
PEDF-R	GGTCCAAGCGAGGGTTGCCC
GAPDH-F	AGCCTCCCGCTTCGCTCTCT
GAPDH-R	CCAGGCGCCCAATACGACCA
